# Late‐onset argininosuccinic aciduria in a 72‐year‐old man presenting with fatal hyperammonemia

**DOI:** 10.1002/jmd2.12251

**Published:** 2021-09-26

**Authors:** Laurent Leuger, Xavier Dieu, Juan Manuel Chao de la Barca, Mikael Moriconi, Guillaume Halley, Xavier Donin de Rosière, Pascal Reynier, Delphine Mirebeau‐Prunier, Chadi Homedan

**Affiliations:** ^1^ Laboratoire de Biochimie et biologie moléculaire, Centre Hospitalier Universitaire d'Angers Angers Cedex 9 France; ^2^ Service de Réanimation Polyvalente et Unité de soins continus, Centre Hospitalier de Cornouaille Quimper Cedex France; ^3^ Service de Médecine Polyvalente Ouest, Centre Hospitalier de Cornouaille Concarneau France

**Keywords:** argininosuccinate lyase deficiency, ASLD, hyperammonemia, urea cycle disorders

## Abstract

Argininosuccinate lyase deficiency (ASLD, MIM **#**207900) is an inherited urea cycle disorder. There are mainly two clinical forms, an acute neonatal form which manifests as life‐threatening hyperammonemia, and a late‐onset form characterised by polymorphic neuro‐cognitive or psychiatric presentation with transient hyperammonemia episodes. Here, we report a late‐onset case of ASLD in a 72‐year‐old man carrying a homozygous pathogenic variant in the exon 16 of the *ASL* gene, presenting for the first time with fatal hyperammonemic coma. This case report shows the need to systematically carry out an ammonia assay when faced with an unexplained coma.

**Synopsis**

We report a late‐onset case of ASLD in a 72‐year‐old man, carrying a homozygous pathogenic variant of the *ASL* gene and presenting with for the first time with fatal hyperammonemic coma.

## 1 INTRODUCTION

**FIGURE 1:**
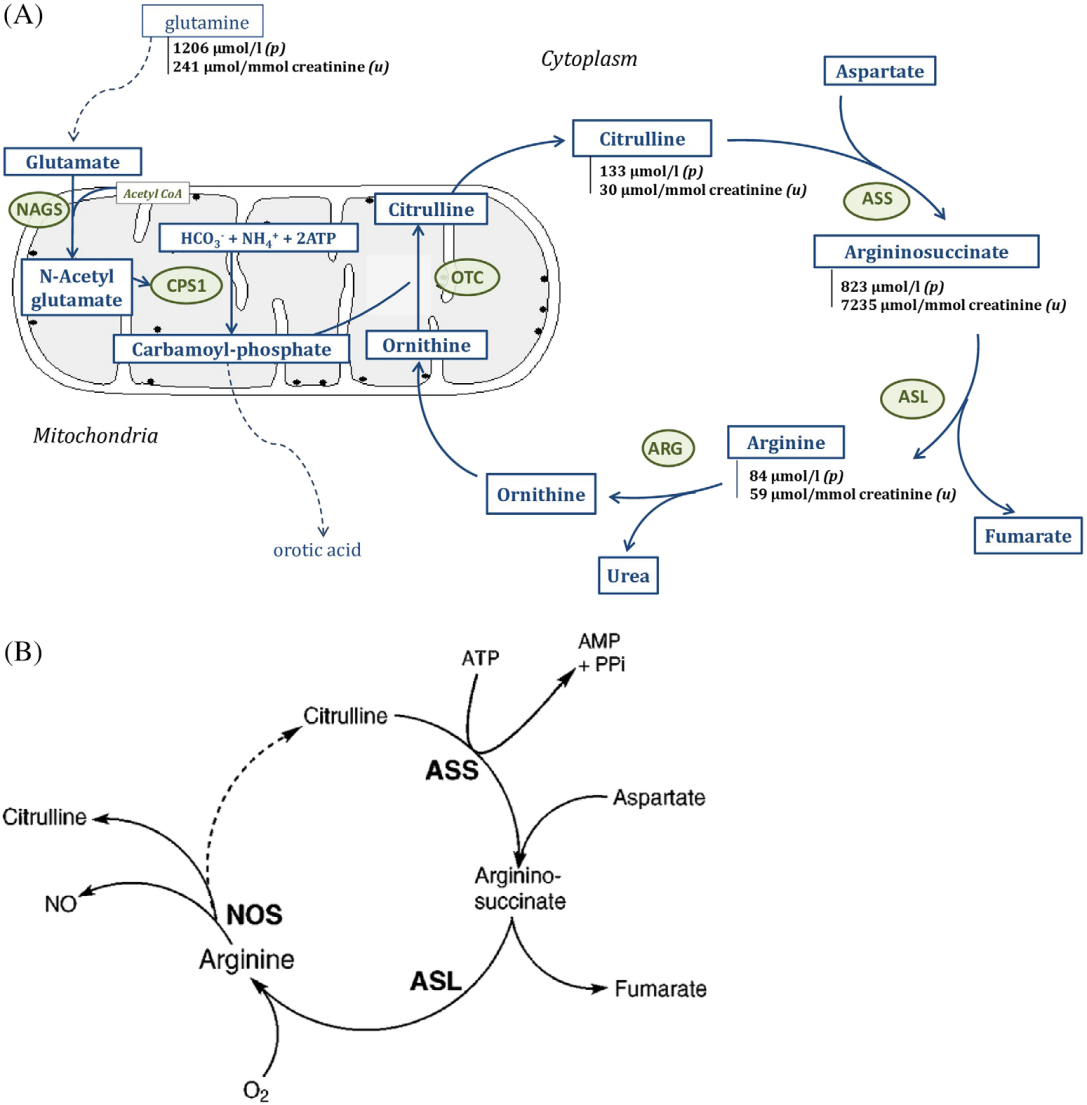
(A) Urea cycle. NAGS: N‐acetylglutamate synthase; CPS 1: carbamoyl phosphate synthetase 1; OTC: ornithine transcarbamylase; ARG: arginase. Patient's parameters in plasma (p) and in urine (u) are marked in black and bold. (B) Citrulline‐NO cycle. ASS: argininosuccinate synthetase; ASL: argininosuccinate lyase; NOS: nitric oxide synthase

Argininosuccinate lyase deficiency (ASLD, OMIM #207900), also known as argininosuccinic aciduria (ASA), is an autosomal recessive disorder first described in 1958.^1^ It is the second most common urea cycle disorder, with a prevalence of 1/70 000.^2^, ^3^ Argininosuccinate lyase (ASL, EC 4.3.2.1) is a cytosolic enzyme expressed in various tissues, including the liver, kidney, brain, and fibroblasts. In the liver, ASL catalyses the fourth step of the urea cycle, enabling the conversion of argininosuccinic acid to fumarate and arginine (Figure 1A). ASL deficiency is responsible for an accumulation of argininosuccinic acid in tissues and excretion of argininosuccinic acid in urine, leading to argininosuccinic aciduria, and a reduction in the synthesis of arginine. Arginine is a precursor for the synthesis of urea and ornithine through the urea cycle and is also an important substrate for many other molecules, such as nitric oxide, polyamines, proline, glutamate, and creatine.

The enzyme defect of this autosomal recessive disorder is due to mutations in the *ASL* gene (OMIM *608310) located in chromosome 7.^4^ More than 154 ASL pathogenic variants have been reported (Human Gene Mutation Database),^5^, ^6^ the phenotypic severity being highly correlated with the residual ASL activity.^7^

Various clinical presentations exist,^8^ but two forms are predominant. The most frequent form is a severe neonatal onset presenting with acute hyperammonemic encephalopathy and hyperglutaminemia during the first days of life, leading to lethargy, drowsiness, refusal to feed, vomiting, and tachypnea with secondary respiratory alkalosis. The second clinical presentation is a more insidious late‐onset form characterised by transient episodes of hyperammonemia, triggered by stressors such as infections, traumas, excessive protein intake, stress or any other cause of increased protein catabolism,^9^ but also intellectual disability, behavioural disorders, and learning difficulties. Therefore, diagnostic wandering is often observed in this form, delaying therapeutic care and worsening the patient's prognosis. Other symptoms are common to both forms and unrelated to the frequency or duration of hyperammonemic crisis, such as neurologic features (attention deficit, hyperactivity, developmental delay, and learning disability), liver disease (hepatitis and cirrhosis), systemic arterial hypertension, and trichorrhexis nodosa.^10^ The treatment is mainly based on a diet excluding proteins of animal origin to reduce ammoniac production.

Neonatal forms have been widely described in the literature.^9^ Paediatric and young adult late‐onset forms, more difficult to diagnose, have been less frequently reported.^11^, ^12^ Very late‐onset forms (50 years old and over) have been rarely described.^13^ Here, we present one such very late‐onset case of ASLD diagnosed for the first time at 72 years old, following a fatal hyperammonemic coma.

## 2 CASE REPORT

The patient was a 72‐year‐old Caucasian male, born from non‐consanguineous parents and childless. He lived alone at home, was under legal protection (curatorship) and received weekly social assistance for disabled people. He stopped his education in primary school. He spent his entire professional career within ESAT (Institution and assistance service for physically/mentally disabled people), working in the maintenance of green spaces. He was not known to follow a particular diet excluding animal proteins. His medical history was an intellectual disability, issues with interpersonal relationships, behavioural disorders and type 2‐diabetes treated with metformin. He had not undergone any hospitalisation until the end of 2019.

He was admitted to the emergency room in November 2019 for a fall followed by an extended period of around 12 h lying on the ground. Neurological examination was normal. The patient was afebrile, with pronounced asthenia, biological inflammatory syndrome (hyperleukocytosis, thrombocytosis, elevated C‐reactive protein 172 mg/L, alpha‐1 protein 6.3 g/L and alpha‐2 protein 13.2 g/L on serum protein electrophoresis), elevated transaminases and slightly elevated AP and GGT (ALAT and ASAT = 3 N, GGT and AP < 2 N). On admission to the emergency room, no disturbance of consciousness was noted during the initial clinical examination, with a Glasgow score of 15. He was transferred to a general medicine department. The trauma assessment of his fall did not reveal any fracture. No infectious call points were found on chest X‐ray and urines. No glycemic imbalance was found in this diabetic patient and a reduced carbohydrate diet with a normal protein intake was put in place. The clinical condition remained stable for a week, before the sudden onset of a hypotonic coma with pyramidal signs (Glasgow Coma Scale = 4), justifying a transfer to an intensive care unit. Glycemia was normal (6.71 mmoL/L). A stroke was suspected but not confirmed by CT scan, while additional investigations (brain MRI, lumbar puncture) could not explain the coma.

Hyperammonemic encephalopathy was then suspected and confirmed by an ammonia assay performed 3 days after coma‐onset, showing a plasma concentration of 167 μmoL/L with a peak value of 1500 μmoL/L (normal plasma level < 50 μmoL/L). The patient also presented with persistent hyperlactatemia (peak value of 3.7 mmoL/L). Shortly thereafter, multiple organ failure appeared with acute renal failure (creatinine 149 μmoL/L, urea 9.25 mmoL/L) and hepatic cytolysis (ASAT 27 N/ALAT 18 N). Ammonia was controlled by hemofiltration and a fasting period relayed by a protein‐free diet (1307 μmoL/L 1 day after the start of hemofiltration, 140 μmoL/L 4 day later). The clinical outcome was unfavourable. Brain death was confirmed by cerebral CT angiography 2 weeks after coma onset.

Metabolic investigation was performed in the days following hospitalisation in the intensive care unit, as soon as hyperammonia was detected. ASL deficiency was suspected from the results of plasma amino acid profile (chromatography of plasma amino acids), showing a high level of argininosuccinic acid at 823 μmol/L (normal plasma level: unrecordable). Citrulline concentration was also elevated at 133 μmoL/L (normal plasma level < 33 μmoL/L) as well as glutamine at 1206 μmoL/L (normal plasma level < 670 μmoL/L), reflecting the significant hyperammonemia. The urinary profile of amino acid (chromatography of urinary amino acids) revealed an elevation of the same analytes: increase excretion of argininosuccinate at level of 7235 μmol/mmol of creatinine (normal urinary level: undetectable), of citrulline at level at 30 μmol/mmol of creatinine (normal urinary level: 4 μmol/mmol creatinine) and of glutamine at level of 241 μmol/mmol of creatinine (normal urinary level < 36 μmol/mmol creatinine). Urine organic acid profile showed an increase in orotic acid at 354 μmol/mmol of creatinine. Orotic acid is a cytosolic metabolite highly specific to urea cycle deficiency, synthesised from carbamoyl phosphate and accumulating into mitochondria in ASLD.

The diagnosis of ASL deficiency was confirmed by whole exome sequencing, revealing a homozygotic missense pathogenic variant in exon 16 of the *ASL* gene (NM_001024944, c.1306C>T, p.R436W). This variant is known to be pathogenic by Clinvar (https://www.ncbi.nlm.nih.gov/clinvar/variation/660049/) and Uniprot databases. It has previously been reported in a compound heterozygous state in the literature but to our knowledge never in a homozygous state. The allele frequency of the variant in the gnomAD whole population database is 0.00002880, with most alleles (5) in European non‐Finnish individuals, two in African/African‐Americans and one in other populations (https://gnomad.broadinstitute.org/variant/7-65557870-C-T?dataset=gnomad_r2_1).

## 3 DISCUSSION

This case‐report presents a very late‐onset occurrence of fatal hyperammonemia in a patient with ASLD. The patient had previously shown symptoms, such as an intellectual disability and a behavioural disorder fitting with the ASLD clinical picture, but the initial diagnosis of ASLD has been missed until this severe metabolic decompensation because of the non‐specificity of these initial symptoms. The mental impairment and behavioural disorder presented by the patient are typical symptoms of late‐onset forms of ASLD.

The variability of ASLD clinical expression depends on ASL residual activity.^14^ Therefore, frequency of hyperammonemic crisis, ammonia levels and the patient's cognitive profile vary according to this residual activity.^14^ The best known and prevalent ASL variants are mostly missense mutations, well correlated with neonatal‐onset forms (p.Arg12Gln, p.Ile100Thr, p.Val178Met, p.Arg186Trp, p.Glu189Gly, p.Gln286Arg, and p.Arg385Cys).^15^ It appears probable that the most deleterious mutations (nonsense mutations with premature stop codon or frame‐shift mutations), leading to a complete loss of enzymatic activity, are correlated with these most severe neonatal onset forms. Few studies have identified highly conserved regions predicted to form ASL's active sites (Arg111, Ser112, Arg113, Asn114, Val117, His160, Gln286 and Lys 288).^15^ Mutations near or within these domains of the protein may cause more severe forms. Our patient's mutation is located on the last exon (16). In the literature, 13 missense variants have been reported within exon 16, three of which were associated with severe forms of ASLD in the homozygous state.^15^ The residue affected by the patient pathogenic variant was previously analysed in silico by molecular modelling using the human ASL protein, showing that it affects the terminal alpha helix of the protein position, which is important for dimerization.^16^ In addition, yeast used as a model to measure ASL activity in vivo showed significant residual activity and allowed to classify this variant as hypomorphic, thus explaining the unusual course of the disease in this case.^17^

Hyperammonemia was probably caused by protein hypercatabolism due to biological inflammatory syndrome associated with the continuation of a normal protein intake. The trigger factors of the decompensation on top of the inflammatory syndrome were likely to be the loss of appetite and poor calorie supply through IV infusion. About half of patients with late‐onset phenotype do not present hyperammonaemia.^18^ Before 2019, there was no documented hyperammonemia crisis in our patient. However, chronic hyperammonemia cannot be excluded, since it could explain the patient's intellectual deficiency, which is the main manifestation of insidious primary urea cycle disorder. Indeed, both chronic and acute hyperammonemia are neurotoxic and could lead to intellectual disability.^19^

Another hypothesis could explain his intellectual impairment. In argininosuccinic aciduria, several works have shown the existence of neurocognitive disorders despite early initiation of therapy and even in the absence of documented hyperammonemia.^9^ Plasma ammonia is not the only toxic compound in ASLD, other neurotoxic mechanisms could lead to mental disorders. ASLD causes a lack of endogenous arginine. Therefore, this amino acid becomes essential and dependent on exogenous intake. NO is synthesised from arginine by NO synthase (NOS) (Figure 1B). In ASLD, arginine deficiency cause NOS uncoupling and NO deficiency^20^ but also the production of reactive oxygen species (O_2_‐, ONOO‐) and nitrotyrosine, a marker of neuronal oxidative/nitrosative stress. This phenomenon, shown by Baruteau and al in an ASL‐deficient mouse, is independent of hyperammonaemia.^21^ Argininosuccinic acid is also neuropathogenic. It interacts with free radicals to form guanidinosuccinic acid, a known cellular and neuronal toxin. This could therefore explain the existence of neurocognitive signs in the absence of hyperammonemia.^22^, ^23^, ^24^

The urinary excretion of orotic acid is markedly increased in many inborn errors of the urea cycle, but typically, the orotic acid excretion is within the normal range in ASLD. However, orotic aciduria could be observed in this condition as shown in our patient.^25^ The impaired recycling of ornithine may contribute to an increase of carbamoyl phosphate leading to overproduction of orotic acid.

In this case, the finding of hyperammonemia was the clue that led to a urea cycle defect being suspected. In practice, every patient with unexplained intellectual disability or a coma of unknown aetiology should have their plasma ammonia measured.

## CONFLICT OF INTEREST

The authors declare that they have no conflict of interest.

## INFORMED CONSENT

All procedures followed were in accordance with the ethical standards of the responsible committee on human experimentation (institutional and national) and with the Helsinki Declaration of 1975, as revised in 2000. Written informed consent for publishing this report was obtained from the patient's cousin (his trusted person).

## AUTHOR CONTRIBUTIONS

Clinical patient care and diagnosis: Mickael Moriconi, Guillaume Halley and Xavier Donin de Rosière. Evaluation/interpretation of metabolic, genetic and radiologic results: Laurent Leuger, Xavier Dieu, Juan Manuel Chao de la Barca, Pascal Reynier, Delphine Mirebeau‐Prunier and Chadi Homedan. Drafting and revision of the manuscript: Laurent Leuger, Xavier Dieu, Juan Manuel Chao de la Barca, Mickael Moriconi, Guillaume Halley, Xavier Donin de Rosière, Pascal Reynier, Delphine Mirebeau‐Prunier and Chadi Homedan. All authors agreed with the final version of the manuscript.

## References

[1] Allan JD , Cusworth DC , Dent CE , Wilson VK . A disease, probably hereditary characterized by severe mental deficiency and a constant gross abnormality of aminoacid metabolism. Lancet. 1958;1:182‐187. 10.1016/s0140-6736(58)90666-4 13503250

[2] Brusilow S , Horwich A . Urea cycle enzymes. In: Scriver CR , Beaudet AL , Sly WS , et al., eds. The Metabolic and Molecular Bases of Inherited Disease. 8th ed. Chapter 85 New York: McGraw‐Hill; 2001:1909‐1963.

[3] Ozben T . Expanded newborn screening and confirmatory follow‐up testing for inborn errors of metabolism detected by tandem mass spectrometry. Clin Chem Lab Med. 2013;51:157‐176. 10.1515/cclm-2012-0472 23183752

[4] Naylor SL , Klebe RJ , Shows TB . Argininosuccinic aciduria: assignment of the argininosuccinate lyase gene to the pter to q22 region of human chromosome 7 by bioautography. Proc Natl Acad Sci U S A. 1978;75:6159‐6162. 10.1073/pnas.75.12.6159 282632 PMC393138

[5] Barbosa P , Cialkowski M , O'Brien WE . Analysis of naturally occurring and site‐directed mutations in the argininosuccinate lyase gene. J Biol Chem. 1991;266:5286‐5290. PMID: 1705937. 1705937

[6] O'Brien WE , McInnes R , Kalumuck K . Adcock MCloning and sequence analysis of cDNA for human argininosuccinate lyase. Proc Natl Acad Sci U S A. 1986;83:7211‐7215. 10.1073/pnas.83.19.7211 3463959 PMC386685

[7] Zielonka M , Garbade SF , Gleich F , et al. Urea cycle disorders consortium (UCDC) and the European registry and network for intoxication type metabolic diseases (E‐IMD) Consortia Study Group. From genotype to phenotype: early prediction of disease severity in argininosuccinic aciduria. Hum Mutat. 2020;41:946‐960. 10.1002/humu.23983 31943503 PMC7428858

[8] Baruteau J , Diez‐Fernandez C , Lerner S , et al. Argininosuccinic aciduria: recent pathophysiological insights and therapeutic prospects. J Inherit Metab Dis. 2019;42:1147‐1161. 10.1002/jimd.12047 30723942

[9] Nagamani SCS , Erez A , Lee B . Argininosuccinate lyase deficiency. In: Adam MP , Ardinger HH , Pagon RA , et al., eds. GeneReviews®. Seattle, WA: University of Washington; 2011:1993‐2020 PMID: 21290785. 21290785

[10] Fichtel JC , Richards JA , Davis LS . Trichorrhexis nodosa secondary to argininosuccinic aciduria: trichorrhexis nodosa. Ped Dermatol. 2007;24:25‐27. 10.1111/j.1525-1470.2007.00327.x 17300644

[11] Osawa Y , Wada A , Ohtsu Y , Yamada K , Takizawa T . Late‐onset argininosuccinic aciduria associated with hyperammonemia triggered by influenza infection in an adolescent: a case report. Mol Genet Metab Rep. 2020;24:100605. 10.1016/j.ymgmr.2020.100605 32435591 PMC7232106

[12] Roze E , Azuar C , Menuel C , Häberle J , Guillevin R . Usefulness of magnetic resonance spectroscopy in urea cycle disorders. Pediatr Neurol. 2007;37:222‐225. 10.1016/j.pediatrneurol.2007.05.003 17765814

[13] Lågas PA , Ruokonen A . Late‐onset argininosuccinic aciduria in a paranoid retardate. Biol Psychiatry. 1991;30:1229‐1232. 10.1016/0006-3223(91)90159-j 1790264

[14] Zielonka M , Garbade SF , Gleich F , et al. From genotype to phenotype: early prediction of disease severity in argininosuccinic aciduria. Hum Mutat. 2020;41:946‐960. 10.1002/humu.23983 31943503 PMC7428858

[15] Balmer C , Pandey AV , Rüfenacht V , et al. Mutations and polymorphisms in the human argininosuccinate lyase (ASL) gene. Hum Mutat. 2014;35:27‐35. 10.1002/humu.22469 24166829

[16] Trevisson E , Burlina A , Doimo M , et al. Functional complementation in yeast allows molecular characterization of missense argininosuccinate lyase mutations. J Biol Chem. 2009;284:28926‐28934. 10.1074/jbc.M109.050195 19703900 PMC2781438

[17] Trevisson E , Salviati L , Baldoin MC , et al. Argininosuccinate lyase deficiency: mutational spectrum in Italian patients and identification of a novel ASL pseudogene. Hum Mutat. 2007;28:694‐702. 10.1002/humu.20498 17326097

[18] Baruteau J , Jameson E , Morris AA , Chakrapani A . Expanding the phenotype in argininosuccinic aciduria: need for new therapies. J Inherit Metab Dis. 2017;40:357‐368. 10.1007/s10545-017-0022-x 28251416 PMC5393288

[19] Gropman AL , Summar M , Leonard JV . Neurological implications of urea cycle disorders. J Inherit Metab Dis. 2007;30:865‐879. 10.1007/s10545-007-0709-5 18038189 PMC3758693

[20] Erez A , Nagamani SCS , Shchelochkov OA , et al. Requirement of argininosuccinate lyase for systemic nitric oxide production. Nat Med. 2011;17:1619‐1626. 10.1038/nm.2544 22081021 PMC3348956

[21] Baruteau J , Perocheau DP , Hanley J , et al. Argininosuccinic aciduria fosters neuronal nitrosative stress reversed by ASL gene transfer. Nat Commun. 2018;9:3505. 10.1038/s41467-018-05972-1 30158522 PMC6115417

[22] Aoyagi K , Shahrzad S , Iida S , et al. Role of nitric oxide in the synthesis of guanidinosuccinic acid, an activator of the N‐methyl‐D‐aspartate receptor. Kidney Int Suppl. 2001;78:S93‐S96. 10.1046/j.1523-1755.2001.59780093.x 11168991

[23] Aoyagi K . Inhibition of arginine synthesis by urea: a mechanism for arginine deficiency in renal failure which leads to increased hydroxyl radical generation. Mol Cell Biochem. 2003;244:11‐15. PMID: 12701804. 12701804

[24] D'Hooge R , Pei YQ , Marescau B , De Deyn PP . Convulsive action and toxicity of uremic guanidino compounds: behavioral assessment and relation to brain concentration in adult mice. J Neurol Sci. 1992;112:96‐105. 10.1016/0022-510x(92)90138-b 1469446

[25] Sandesh C , Nagamani S , Erez A , Lee B . Argininosuccinate lyase deficiency. Genet Med. 2012;14:501‐507. 10.1038/gim.2011.1 22241104 PMC3709024

